# An Acetylcholinesterase-Based Chronoamperometric Biosensor for Fast and Reliable Assay of Nerve Agents

**DOI:** 10.3390/s130911498

**Published:** 2013-08-30

**Authors:** Miroslav Pohanka, Vojtech Adam, Rene Kizek

**Affiliations:** 1 Faculty of Military Health Sciences, University of Defence, Trebesska 1575, CZ-500 01 Hradec Kralove, Czech Republic; 2 Karel English College in Brno, Sujanovo namesti 356/1, CZ-602 00 Brno, Czech Republic; 3 Central European Institute of Technology, Brno University of Technology, Technicka 3058/10, CZ-616 00 Brno, Czech Republic; E-Mails: vojtech.adam@mendelu.cz (V.A.); kizek@sci.muni.cz (R.K.); 4 Department of Chemistry and Biochemistry, Faculty of Agronomy, Mendel University in Brno, Zemedelska 1, CZ-613 00 Brno, Czech Republic

**Keywords:** biosensor, acetylcholinesterase, sarin, tabun, soman, VX, inhibitor, screen printed electrode, voltammetry, amperometry

## Abstract

The enzyme acetylcholinesterase (AChE) is an important part of cholinergic nervous system, where it stops neurotransmission by hydrolysis of the neurotransmitter acetylcholine. It is sensitive to inhibition by organophosphate and carbamate insecticides, some Alzheimer disease drugs, secondary metabolites such as aflatoxins and nerve agents used in chemical warfare. When immobilized on a sensor (physico-chemical transducer), it can be used for assay of these inhibitors. In the experiments described herein, an AChE- based electrochemical biosensor using screen printed electrode systems was prepared. The biosensor was used for assay of nerve agents such as sarin, soman, tabun and VX. The limits of detection achieved in a measuring protocol lasting ten minutes were 7.41 × 10^−12^ mol/L for sarin, 6.31 × 10^−12^ mol/L for soman, 6.17 × 10^−11^ mol/L for tabun, and 2.19 × 10^−11^ mol/L for VX, respectively. The assay was reliable, with minor interferences caused by the organic solvents ethanol, methanol, isopropanol and acetonitrile. Isopropanol was chosen as suitable medium for processing lipophilic samples.

## 1. Introduction

Nerve agents are a group of neurotoxic compounds having common molecular mechanism of action. They are irreversible inhibitors of the enzyme acetylcholinesterase (AChE; EC 3.1.1.7). It is noteworthy that the enzyme plays a crucial role in cholinergic neurotransmission [1,2]. The nerve agents are probably the most toxic chemical substances used for chemical warfare. Their median lethal doses extrapolated for humans are much lower than the doses for blister or blood agents [1,3]. Organophosphate and carbamate pesticides have similar mechanisms of action, because they can also inhibit AChE by irreversible (organophosphates) and pseudo-irreversible (carbamates) mechanisms of action [2,4].

The fact that AChE can be inhibited by neurotoxic compounds is not only a negative phenomenon. Firstly, many drugs act as inhibitors of AChE. Secondly, the phenomenon can be used in analytical chemistry. The activity of AChE can easily be measured using disparate colorimetric, fluorimetric and voltammetric protocols [5]. When the measurement is performed *in vitro*, it can be used for an assay of the aforementioned inhibitors [6]. The assay can be done using standard cuvettes, microplates, *etc.* Besides the aforementioned, it is well suited for the construction of biosensors that are miniaturized devices containing a physico-chemical transducer in a tight junction with a biorecognition element, in this case represented by the AChE [7,8].

The intention in the present experiments was to develop a biosensor to assay highly toxic nerve agents. For this purpose, a novel immobilization procedure based on capturing of AChE into a gelatin membrane and consequent stabilization by glutaraldehyde was chosen. Nerve agents are not easy to detect prior to the appearance of human casualties when misused in chemical warfare or chemical terrorism. Experiences from the Tokyo subway attack point to the necessity of fast countermeasures [9,10]. Detection of the agents is needed for initiation of the provision of suitable prophylactic and therapeutic care. We have thus aimed our efforts at constructing a cheap and reliable device suitable for both laboratory and field assays.

## 2. Experimental Section

### 2.1. Preparation of the Biosensor

Lyophilized electric eel AChE (specific activity 16.7 μkat/mg protein) was purchased from Sigma-Aldrich (Saint Louis, MO, USA). The enzyme was dissolved in phosphate buffered saline prior to assay. In order to specify and adjust the amount of enzyme, AChE activity was measured by a modified Ellman's method in a way introduced in previous papers [11–13].

**Figure 1.**
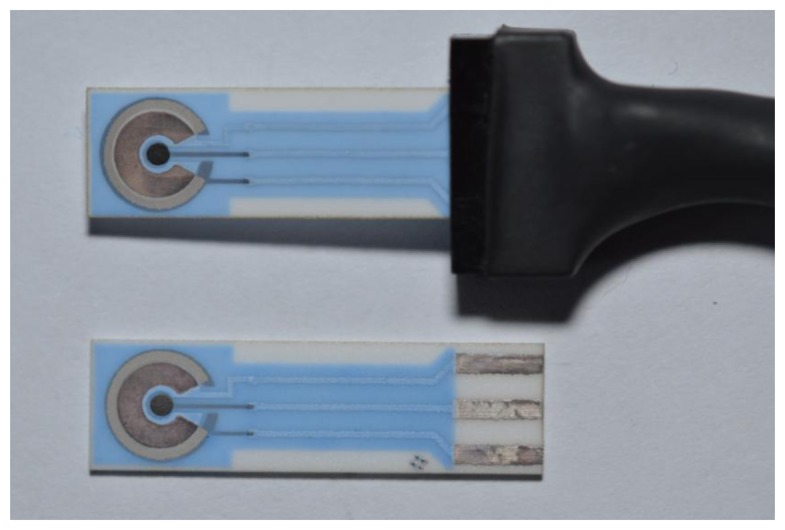
Screen printed sensors used in the experiments. In the upper part, sensor in a plastic holder is depicted. In the bottom part, a naked sensor is shown. Electrodes are on the left side of the picture, output contacts on the right.

Screen printed sensors were received from BVT Technologies (Brno, Czech Republic). The sensors are depicted in Figure 1. We chose sensors sized 25.4 × 7.3 × 0.6 mm containing a central platinum dot shaped working electrode with diameter 1 mm, platinum auxiliary and a reference electrode consisting of silver covered with silver chloride. We chose the platinum working electrodes in compliance with our good previous experience working with this material and data found in the literature [14–17]. The working electrode was covered with 10 μL of a solution containing AChE 20 U for 1 mmol/L acetylthiocholine as a substrate, gelatin 1 mg/mL (Sigma-Aldrich) and 1% (*w*/*v*) glutardialdehyde (Sigma-Aldrich). The solution was dried at laboratory temperature overnight. After that, surface of the biosensor was rinsed with phosphate buffered saline and dried at laboratory temperature. The freshly prepared biosensors were used immediately or kept stored at 4 °C until needed.

### 2.2. Nerve Agent Assay

**Figure 2.**
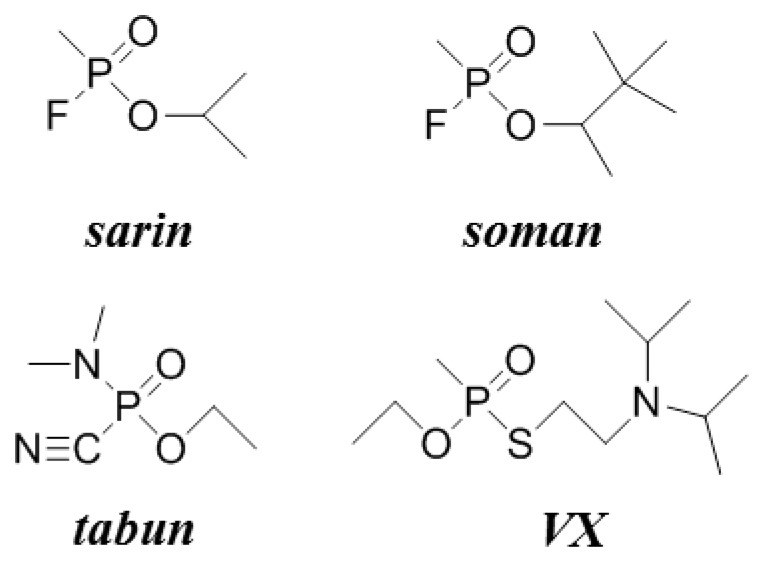
Structures of nerve agents used for the experiment.

Sarin, soman, tabun and VX were used as analytes inhibiting AChE without any pre-treatment. The structures of the tested nerve agents are shown in Figure 2. The compounds were received from the former Military Technical Institute (Brno, Czech Republic) in the mid-1990s. Purity of nerve agents was at least 95% (*w*/*w*). Storage and manipulation of the nerve agents was accredited by the State Office for Nuclear Safety, an institution representing the Organization for the Prohibition of Chemical Weapons. All experiments with the nerve agents were done in a specialized laboratory at the Faculty of Military Health Sciences, University of Defence, Hradec Kralove, Czech Republic. Chemical boxes with carbon powder filters at the exhaust were used for all manipulations involving nerve agents. Materials and remnants of nerve agents solutions were decontaminated in 1 mol/L NaOH with 10% ethanol (for sarin, soman, tabun) or 30% calcium hypochlorite (for VX). The decontamination solutions were allow to act at least overnight.

The aforementioned nerve agents were dissolved in isopropanol in a calibration scale 10^−4^, 10^−5^, 10^−6^, 10^−7^, 10^−8^, 10^−9^, 10^−10^, and 10^−11^ mol/L. Pure isopropanol was used as the control. The biosensor was fixed in a holder, linked by a rubber covered cable with PalmSens (PalmSens BV, Houten, The Netherlands) device and located within a 1 mL sized cuvette. The whole measurement was controlled using PSLite 1.8 (PalmSens BV) software installed on a computer. The measurement had following steps:

- 800 μL of phosphate buffered saline was injected into the cuvette.
- 100 μL of the sample to be tested was added to the cuvette and allowed to incubate for 10 min.
- Reaction was started by an addition of 100 μL of 10 mmol/L acetylthiocholine and the resulting current was measured using chronoamperometry at a given potential of 640 mV. This potential was used in compliance with the former experiments [13].

### 2.3. Data Processing

All samples were assayed in tetraplicate and standard deviations were calculated for every point of the calibration scale. Experimental data were processed in the Origin 8 software (OriginLab Corporation, Northampton, MA, USA). The calibration curves were fitted by Hill equation with coefficient of cooperativity adjusted up n = 1. Limit of detection was calculated from the confidence interval (95%) in a calibration plot in compliance with quoted papers [18,19]. The limit of detection calculation was based on determination of blank value crossing with the upper confidence interval. The chosen calculation of limit of detection is advantageous for a standard calculation of signal to noise ratio equal to three because the used confidence intervals take the whole data set into account. The expressed limits of detections and concentrations in the figures respond to final concentration of sample in the cuvette unless stated otherwise.

## 3. Results and Discussion

**Figure 3.**
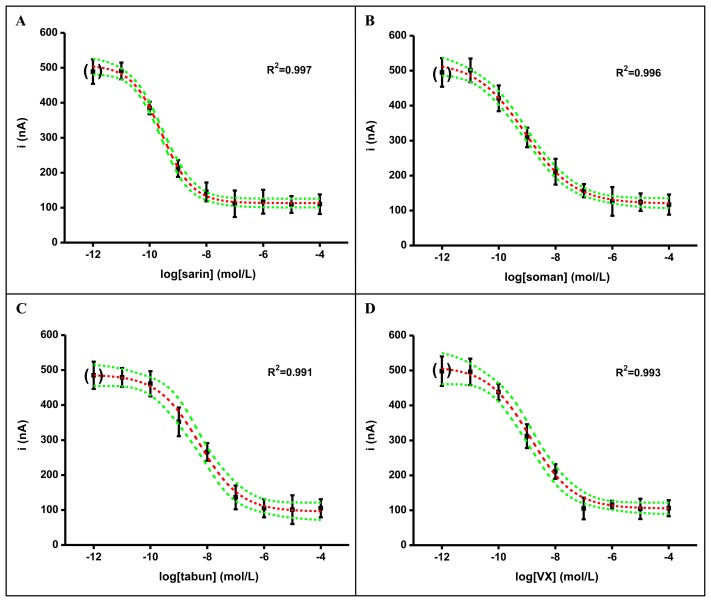
Calibration of AChE based biosensors to the tested nerve agents sarin (**A**), soman (**B**), tabun (**C**) and VX (**D**). Error bars indicate standard deviation for n = 4 and green lines indicate the 95% confidence interval. The point in brackets was achieved by a blank assay.

In the here described experiments, immobilization based on capturing AChE into a gelatin membrane with stabilization by glutaraldehyde was done. The freshly prepared biosensors were successfully used for assay of the nerve agents. Calibration curves are shown in Figure 3 for sarin (A), soman (B), tabun (C) and VX (D). Limit of detections for the nerve agents were in a quite narrow range. The best limits of detection were achieved for sarin and soman. The worst limit of detection was received for tabun. The exact limits of detection were as follows: sarin 7.41 × 10^−12^ mol/L, soman 6.31 × 10^−12^ mol/L, tabun 6.17 × 10^−11^ mol/L, VX 2.19 × 10^−11^ mol/L. When considering the limits of detection reached, we have to emphasize that ability to detect low amounts of nerve agents is necessary when a warning about the presence of nerve agents should precede development of manifestations of toxicity. In an example, the estimated median lethal concentration and time is 55 mg-min/m^3^ for sarin and 15 mg-min/m^3^ for VX [1,3]. The AChE based biosensor is expected to be used as a tool for early warning during chemical warfare or terrorist menace. From this point of view, short time for one analysis, low detection limit, low volume of sample and simple manipulation with the device is needed [20]. When considering the reported toxicity of the assayed nerve agents and the found limits of detection, we could not prove any correlation between the two values. Though the upper toxicity is reported for tabun and VX [1,4], the biosensor has a lower limit of detection for the less toxic sarin and tabun. This is not, however, surprising. The ability to cross a membrane with immobilized AChE is necessary for an easy assay. Compared to this, the distribution and elimination processes in the human body are not identical to the environment in the reaction cell and membrane on the biosensor surface.

The results indicate a substantial advantage of AChE-based biosensors to assay nerve agents without the need for any expensive device or complicated protocol. Though the biosensors have some disadvantages, especially in their unsuitability for automated long term monitoring, the assays can be easily performed under field conditions and they are quite cheap compared to physical detectors such as mass spectrometers [21,22]. Nerve agents can be also detected by colorimetric devices with scoring by a naked eye [23]. Paraoxonase-based colorimetric biosensors are another option [24,25]. Overall the simplicity and easy interpretation of data is a major advantage of the colorimetric devices. On the other hand, the electrochemical protocol proposed here allows one to reach lower limits of detection. Electrochemical detection of nerve agents can be further used for miniaturization and partially automated assay procedures [26]. The limits of detection reached by our biosensor are close to the limit of detection of organophosphate and carbamate pesticide detections described in papers devoted to AChE-based electrochemical biosensors [27], bulk acoustic biosensors [25] and surface plasmon resonance biosensors [28]. Arduini and co-workers used a butyrylcholinesterase based electrochemical biosensor and analysed sarin and VX with detection limits of 12 and 14 ppb [29]. Schulze and co-workers reached even lower limits of detection than we did when they used a protein engineered AChE [30]. In an example, Schulze and co-workers detected pirimiphos in a concentration of 3.5 × 10^−12^ mol/L. Their results are also interesting due to fact that they analysed pesticides with lower affinity (lower bimolecular rate constant) toward the enzyme. Comparing with their work, we analysed highly reactive compounds. Limitations for successful assay of nerve agents result from the lack of ability to diffuse through a membrane rather than the affinity toward AChE, which is high enough.

It should be emphasized that the biosensor was used for assay of highly toxic nerve agents that are not readily available to most researchers. In the current scientific literature, AChE-based biosensors are dominantly used for the more widely available pesticides. The results presented here are unique for that reason. Moreover, an immobilization procedure using gelatin in combination with gluataraldehyde stabilization was used. The biosensor construction procedure was successful and worthy of use as a platform for standard production of biosensors.

The AChE-based biosensor is not a selective analytical tool. In the assay, all neurotoxic compounds acting as AChE inhibitors, including some Alzheimer disease drugs, carbamates and oxoforms of organophosphate pesticides can be assayed and the compounds will be active in the assay once presented in a sample. On the other hand, these compounds cause similar toxicological manifestations as the nerve agents and they are also harmful for humans when present in high levels [2,9,31–33]. Organic solvents can be considered as more serious interference problem as they can be anticipated in a sample [34,35] and they are not toxic enough to be considered as a menace. Ethanol and methanol are typical examples of such organic solvents [36]. Immobilization of AChE causes its stabilization and better resistance to organic solvents than typical for the free enzyme [37]. It is a problem because the solvents can be easily considered as suspicious samples or be used for processing samples such as in the extraction of aflatoxins that inhibit AChE as well [38,39]. We tested the interference of ethanol, methanol, isopropanol, and acetonitrile using the prepared biosensors. This was tested when the organic solvent when either applied as a pure sample (100 μL) resulting in a final concentration in the cuvette of 10% (*v*/*v*) and then with a double volume of sample (200 μL) by lowering the volume of g phosphate buffered saline from the original 800 to 700 μL. Percent of inhibition was calculated using a control measurement when phosphate buffered saline was used as a sample. The results are listed in Table 1. We can see that isopropanol caused the lowest inhibition and it appear to be the best for sample processing. Ethanol, methanol and acetonitrile can cause significant inhibition. However, the assay of nerve agents is still functional as there remains a lot of AChE activity on the biosensor. Pertinent increase of sample volume could lead to interference of organic solvent so a maximum sample volume of 100 μL is recommended for the 1 mL cuvette. The findings correlate well to results from known papers [5,13,37].

## 4. Conclusions

We can conclude that the prepared biosensor is suitable for a routine, low cost field assay of nerve agents. There is no need to perform any elaborate manipulation of the samples. The biosensor is reliable, with quite low sensitivity to interference. In the assay, the overall costs are low and the biosensor seems to be amenable to large-scale production.

## Figures and Tables

**Table 1.** Interference of organic solvents expressed as a percent of initial signal where no solvent is applied.

**Final concentration in the cuvette (v/v)**	**Ethanol**	**Methanol**	**Isopropanol**	**Acetonitrile**
10%	31.6 ± 3.8	37.2 ± 2.7	17.4 ± 2.1	29.8 ± 3.5
20%	49.8 ± 4.1	45.6 ± 3.9	34.9 ± 5.6	51.4 ± 5.2
